# The public health role of caseloading midwives in advancing health equity in childbearing women and babies living in socially deprived areas in England: The Mi-CARE Study protocol

**DOI:** 10.18332/ejm/146012

**Published:** 2022-04-04

**Authors:** Charlotte E. Clayton, Ann Hemingway, Mel Hughes, Stella Rawnson

**Affiliations:** 1 Department of Medical Sciences and Public Health, Faculty of Health and Social Sciences, Bournemouth University, Bournemouth, United Kingdom; 2 Department of Social Sciences and Social Work, Faculty of Health and Social Sciences, Bournemouth University, Bournemouth, United Kingdom; 3 Department of Midwifery and Health Sciences, Faculty of Health and Social Sciences, Bournemouth University, Bournemouth, United Kingdom

**Keywords:** qualitative research, public health, social determinants of health, midwifery continuity of care, constructivist grounded theory, health equity

## Abstract

This article outlines the protocol for a qualitative Constructivist Grounded Theory study, examining the public health role of caseloading midwives working in a continuity model of care in areas of urban social deprivation. The study is currently being conducted in a city in the south of England during the COVID-19 pandemic. Focusing specifically on the Social Determinants of Health impacting women and babies in this context and from the perspectives of women themselves, the study is developing a theoretical framework examining the actions caseloading midwives take in response to these determinants and how these actions contribute to advancing equity and equality for women and babies at increased risk of adverse perinatal outcomes. Examining and integrating the experiences of women and midwives from a Constructivist Grounded Theory perspective, the study findings will inform current NHS maternity policy and contribute to our understanding about the social processes and mechanisms underpinning the known benefits of midwifery continuity of care models in different contexts.

## INTRODUCTION

Living in a deprived area is known to negatively impact on health outcomes and quality of life across the life course^1,2^. The Social Determinants of Health (SDH), which describe the contexts and conditions people are born, grow, work, live, and age in, are recognized as having a significant impact on health differences seen between the richest and poorest^3,4^. The SDH intersect with other determinants such as gender, race, disability, and culture, all of which affect differences in health and wellbeing^5^. There are also clear socioeconomic disparities seen in levels of avoidable disease in women, affecting cardiovascular and respiratory health^5^. Life expectancy has also declined for women living in the most deprived areas in England, for the first time in more than a hundred years^5^. Furthermore, the COVID-19 pandemic further amplified historical socioeconomic and racial inequalities throughout the United Kingdom (UK), especially for families from minority ethnic backgrounds and those living in the most deprived areas^6,7^.

Pregnant women living in the most deprived areas in England are more likely to enter pregnancy with pre-existing co-morbidities such as raised BMI, poor nutrition, hypertension, mental health conditions, learning disabilities, diabetes, and undiagnosed medical disorders^8^. Poor physical health often intersects with other social risk factors as outlined in Table 1, which increase incidences of intervention, adverse perinatal outcomes, and poor experiences of maternity care^8^. In addition, women living in the most deprived areas are 50% more likely to experience a stillbirth or neonatal death^9^, and also have a threefold chance of dying in pregnancy compared to women living in the least deprived neighbourhoods^8^. Babies are also more likely to be born prematurely and with a low birth weight, causing significant health disparities including developmental delay, learning disabilities, lung disease, visual and hearing problems, as well as other health complications^10^. These factors contribute to increased costs to health and social care systems, economies, and society as a whole^10^.

**Table 1:** Summary of social risk factors experienced by some women living in deprived areas, associated with poor perinatal outcomes and experiences of care

*Women at increased social risk of poor perinatal outcomes and who particularly benefit from midwifery-led continuity of care^1-3, 6-9^*
Black, Asian, and Minority ethnicity women
Female genital mutilation
HIV positive status
Housing insecurity and homelessness
Non-native language speakers
Not in education, training, or employment
Perinatal mental health conditions
Physical/emotional and/or learning disability
Poverty and deprivation
Refugees/asylum seekers
Sex workers
Single women
Social isolation
Substance and/or alcohol dependency
Travelling community
Victims of domestic abuse, trafficking, modern slavery, war, conflict etc.
Safeguarding concerns
Social service and/or criminal justice system involvement
Young women

Whilst healthcare can never fully compensate for the impact of socioeconomic health disparities, maternity care provided by the National Health Service (NHS) in the UK, is in a unique position to uncover, understand, and take action on maternal and infant health inequalities^11^. Individual midwives, maternity services, and maternity systems by the very nature of being involved throughout the pre-pregnancy, pregnancy, intrapartum, and postnatal periods (hereafter referred to as the childbearing continuum), have diverse public health responsibilities which can influence health inequities^12^. There is a strong international consensus that continuity of midwife-led care significantly contributes to increased perinatal survival whilst also mitigating against the unnecessary use of medical interventions and resources^13^. In 2015, a seminal Cochrane review by Sandall et al.^14^ found Midwifery Continuity of Carer (MCoC) models for women at ‘low’ and ‘high’ risk of complications (but not currently experiencing complications), were the only complex intervention associated with a reduction in preterm birth and improved perinatal survival. Women who received planning, organization, and delivery of care by a named midwife or small team of caseloading midwives throughout the childbearing continuum, were on average 24% less likely to have a preterm birth and 19% less likely to experience pregnancy loss before 24 weeks, compared to women receiving standard care, where they were likely to see different midwives at each appointment^14^. Women also reported consistently higher satisfaction with their care when receiving care from a known midwife. However, outcomes and experiences of care for ‘mixed-risk women’ in different contexts, such as women living in deprived areas, and ‘why’ and ‘how’ these might be so were not included. Recommendations from the Cochrane review^14^ and subsequent international consensus by Kennedy et al.^15^, called for research to prioritize asking different questions using established and transformative methodologies which capture the underlying social processes of human interaction that enhance or constrain the wellbeing of women and their families. Moreover, research needs to prioritize what matters most to women and take account of the mechanisms underpinning models of maternity care in different contexts that influence outcomes.

In the study described in this protocol, mechanisms refer to the social processes shaping how MCoC caseloading midwives interpret, respond, and act on the SDH impacting on women’s lives. These influence decisions, which in turn can impact health equity in a specific context. Context refers to the environment in which the MCoC caseloading model functions, which influence mechanisms to affect outcomes and experiences. Lastly, outcomes refer to the consequences of the actions in this specific context.

The understanding of mechanisms impacting the effectiveness of MCoC models in different contexts was examined in a grounded theory study by Griffiths^16^, which explored caseloading midwives’ experiences of caring for women living in areas of high deprivation in New Zealand. The core theoretical framework ‘staying involved, because the need seems so huge’, explained the significance of the trusting relationship developed between women and midwives and the intensity of involvement required to meet women’s needs^16^. Midwives provided what Griffiths coined as being ‘total midwifery care’, which extended beyond the provision of routine clinical care to include a focus on addressing the SDH. Routine clinical care alone was unable to address the social complexities women faced, which included food poverty, housing insecurity, high rents, low-paid insecure work, and so forth. Midwives were unwavering in their support, often going above and beyond their duties. Midwives provided advocacy, support, and navigation for women in the maternity care system, sometimes using their own transport to help women attend appointments with partner organizations such as the police, social care, and housing. Some midwives even reported buying things for women and providing practical help around the house. Midwives believed their support enhanced women’s lives, increased engagement, and impacted outcomes: hence staying involved because the need seemed so huge^16^. This is one of only a few studies to explore the specific actions midwives take to address the SDH as part of their public health role and explains mechanisms involved in this context which influenced outcomes and women's experiences. However, Griffiths^16^ did not examine the experiences of women receiving care from midwives. The study outlined in this protocol will address this gap, examining the experiences of women and midwives to develop a theoretical framework in a UK maternity context.

Recent research conducted by Rayment-Jones et al.^17-21^ and Fernandez Turienzo et al.^22^ is at the forefront of developing our understanding of the social processes and mechanisms underpinning MCoC models in a UK context. The influential program of research by Rayment-Jones et al.^17-21^, examined the effectiveness of two specialized models of MCoC for women with social risk factors in areas of high deprivation in London. The research employed realist evaluation methodology, an approach used to evaluate how, for whom, and in what circumstances complex interventions like MCoC model work. The study assessed two place-based MCoC models, one community and one hospital-based team. The research identified a complex, interconnecting web of system resource and individual response mechanisms working ‘behind the scenes’^20^. The quality of the trusting relationships midwives built with women enabled them to guide women through an unfamiliar maternity care system. Midwives working in the community-based model were more attuned to women’s wider needs, which was often facilitated by the flexibility and place of appointments. This included seeing women in their own home environments to develop a better understanding of the context and conditions of women’s lives. Midwives were able to act quickly on their professional curiosities and abnormal findings and had increased knowledge of locally available support. Interesting mechanisms were also identified when discussing women who had social care involvement. Midwives revealed multiple strategies and resources they used to advocate and help women regain trust in the system. Women responded with increased feelings of safety and trust in their maternity care, improved help-seeking behaviors, behavior change, and disclosure of the social complexities they faced. This in turn improved engagement with services in the local community, which created more opportunities for women to form supportive social networks. Future research, which examines the complexity of mechanisms underpinning community-based care and how they might lead to improving equity in deprived communities is recommended^21^.

A randomized controlled trial (RCT) by Fernandez Turienzo et al.^22^, evaluated a specialist MCoC model for women at increased risk of preterm birth in London. Findings from the pilot RCT found those accessing hospital-based care in the intervention group were at increased risk of preterm birth compared to women in the standard care group accessing community-based MCoC, demonstrating the protective nature of community midwifery-led care. Recommendations for future research included examining the influence of perceptions of trust, safety, quality, anxiety, stress, coordination, referral, and engagement. Moreover, research should explore the determinants affecting women’s lives and the subsequent actions midwives take to address these as part of their public health role.

Evidence from both studies has been integrated into latest maternity guidance in England^11,12^, demonstrating the significance of asking ‘how’ and ‘why’ improvements in outcomes are seen, thereby informing implementation of effective and sustainable models in NHS maternity services^23^. The ‘Delivering Midwifery Continuity of Care at full-scale’^11^ and ‘Equity and Equality’^12^ strategies build on the commitments of the ‘Better Births’ report published in 2016^24^, which aimed to deliver transformation across maternity services in England, to make care safer, more personalized and more equitable. These latest recommendations take account of concerns raised by stakeholders about the challenges of implementation during the COVID-19 pandemic and the need for sufficient resources and support to be successful in delivering MCoC as the default model of care offered to all women^11^. As outlined in the priorities and operational planning guidance for the NHS in 2022/2023, providers across England should put in place the building blocks for a phased implementation of MCoC teams to ensure that most (>51%) women from Black, Asian, and Mixed ethnicity backgrounds and women from the most deprived areas receive MCoC by March 2022. This is with a view to meeting the commitments set out in the NHS Long-Term Plan^26^, which drew on evidence from Homer et al.^27^, demonstrating improved outcomes for women from Black, Asian, and Mixed ethnicity backgrounds. The commitment is such that 75% of women from these ethnic backgrounds and those from the most deprived areas should receive MCoC by March 2024^26^. This calls for care to be universal, delivered at pace, scale and intensity proportionate to the level of disadvantage, which is known as ‘proportionate universalism’^3^. To do this it is essential for maternity services, and at the micro level, midwives, as part of their public health roles, to respond to each woman’s unique health and social situation, with increasing support as inequalities increase so that care is personalized and equity and equality is improved^12^. The specific actions and mechanisms leading to improved equity for women and babies living in deprived areas are not clearly defined. Moreover, there is a clear need to improve our understanding of the public health role of caseloading midwives working in these contexts, which the study reported in this protocol aims to address.

The way in which MCoC midwives engage with and take action on the SDH and the demands on their role in different contexts are also not fully understood^28^. The scope of midwives’ public health role is broad, and the demands and expectations placed on their education, knowledge, and skills in health education, health promotion, and health protection are growing^29^. In 2013 the Royal College of Midwives^30^ (the professional trade union for midwives and maternity support workers in the UK), contributed to an analysis of the increasing demands on the public health roles of healthcare professionals in the UK. They identified the critical need for midwives to be adequately mandated, trained, and supported by education providers and NHS maternity services to address the SDH as part of their role. Practical steps midwives can take such as gathering data, taking action, providing information, and evaluation were presented, but how midwives achieve this in different contexts is left open to interpretation. In 2019 the Nursing and Midwifery Council (NMC), (the professional regulator for nurses and midwives in the UK), published the latest standards of proficiency for midwives in light of the changing contexts in which midwives work^29^. This included a significant focus on their public health contribution. Midwives make a vital contribution to the quality and safety of maternity care and midwives are expected to make an ‘important contribution to population health, understand social and health inequalities, and how to mitigate them through good midwifery care’^29^. ‘Good midwifery care’, as evidenced by Fernandez Turienzo et al.^28^, Griffiths et al.^16^, and Rayment-Jones et al.^18^, goes beyond routine clinical care and highlights the diversification and expertise of midwives within the wider interdisciplinary and multi-agency team.

To raise awareness and refine the scope of MCoC caseloading midwives’ contribution to public health, the study outlined in this protocol builds on these critical knowledge gaps. The research is currently taking place in a city in the south of England and is in the process of developing a theoretical framework using Constructivist Grounded Theory (ConGT) methods, to explain the mechanisms/actions midwives take in response to the SDH affecting the lives of women and their babies living in deprived areas. The study is focusing specifically on the SDH impacting women and babies in this context, from the perspectives of women themselves. In addition, the study is examining the public health role of midwives from the perspectives of women and midwives participating in the study. Integrating the perspectives of both women and midwives into the final theoretical framework, will provide information on what affects women most and what matters to them, whilst also understanding how midwives respond to social complexity. Moreover, this research is timely and will inform the implementation and sustainability of MCoC models in different contexts to advance equity. At the time of writing and to the best of our knowledge, this is the first study to develop a theoretical framework to explain this phenomenon by interviewing midwives and women during the COVID-19 pandemic.

## METHODS

### Constructivist Grounded Theory

This qualitative study is informed by the ConGT approach to grounded theory, develop by Charmaz^31,32^, and is developing a contextually sensitive theoretical framework informed by the perspectives of women who have received or are currently receiving MCoC care and caseloading midwives themselves. ConGT is suitable for research which aims to develop theoretically generalizable explanations of social processes and mechanisms and provides a framework on which to construct rich, innovative, data-driven theory, grounded in the experiences of participants. Charmaz uses the term ‘constructivist’ to acknowledge subjectivity and the researcher’s own influence and involvement in the construction of knowledge^32^. As an interdisciplinary team of academic health and social care professionals the reflexive nature of Charmaz’s ConGT is fitting.

**Figure 1:**
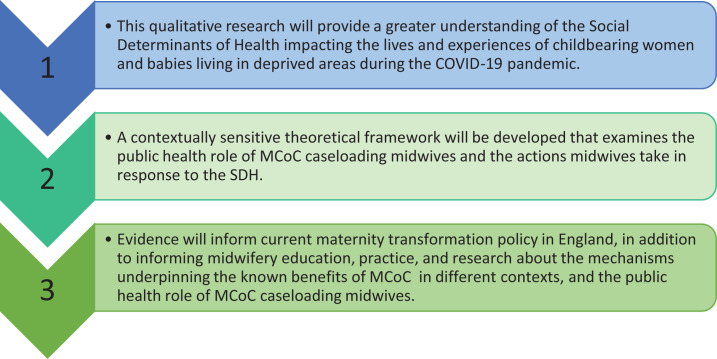
Research outcomes

ConGT is also useful when little is known about an area of interest and an overt focus on social processes is needed^32^. The aim of this study is to develop an understanding that is pragmatic whilst being grounded in the social processes identified by participants. The resultant theory will integrate the experiences of women and midwives. Subsequently there are two sets of research questions, outlined in Table 2 and the intended outcomes of the research are outlined in Figure 1.

**Table 2:** Research questions

**Research questions for childbearing women**
What social issues impact the daily lives of women and babies living in deprived areas, and how do they manage and overcome them?
What do women perceive caseloading midwives’ public health role to be, and how do they experience the care and support they receive?
**Research questions for MCoC caseloading midwives**
What do midwives know about the Social Determinants of Health impacting the lives of women and babies living in deprived areas?
How do midwives engage with and take action on the Social Determinants of Health as part of their public health role to advance health equity?

### Co-production

Each stage of the research design was co-produced and refined as a result of a series of engagement and advisory sessions with midwives, stakeholder organizations, user engagement groups, and women living in the study setting. Consulting the expertise of women with lived experience greatly improved the study’s overall quality, applicability, internal consistency, and relevance for the local target population.

### Research setting

This is a single center study based in a city in the south of England. The study site is an NHS maternity service provider with approximately 5900 births per annum, serving a large multi-cultural, socio-economically diverse childbearing population. Alongside standard maternity care, where women see a number of community and hospital-based midwives throughout the childbearing continuum, the study site has three, well-established mixed-risk, MCoC caseloading teams based in the most deprived areas, which form the focus of this study. At the time of writing, teams provide care based on the presence of known social risk factors as they do not have capacity to provide care to all women living in the deprived areas until more teams are rolled out across the service. These criteria include women from minority ethnic backgrounds. The provider is also the regional tertiary referral center for maternal and fetal medicine.

The study has been designed to comply and adapt at pace with local and national COVID-19 restrictions. Data collection methods and access to the field were adapted as appropriate.

### Eligibility criteria

Tables 3 and 4 detail inclusion and exclusion criteria for each participant group. Individual eligibility is checked by the research team before proceeding to the consent process.

**Table 3:** Inclusion and exclusion criteria for participants in the ‘childbearing women group’

**Inclusion criteria**
Women who are currently receiving maternity care from a MCoC caseloading team in the study setting
OR have previously received care from a caseloading team within the last five years
AND are aged 16 years or older
AND can speak English
AND can independently provide their informed consent.
**Exclusion criteria**
Women who have not received care from one of the MCoC caseloading teams
OR gave birth more than five years ago
OR are below the age of 16 years
OR are known by the lead researcher
OR cannot speak English (this is due to a lack of resources to fund translation services)
OR had their maternity care with a different provider
OR lack capacity to provide their informed consent (i.e. women in labor or women with significant learning disabilities requiring a formal mental capacity assessment)

**Table 4:** Inclusion and exclusion criteria for participants in the ‘midwives' group’

**Inclusion criteria**
Midwives who work in the MCoC caseloading teams in the study setting
OR have previously worked in the local caseloading teams
OR are in senior roles providing wider support to the caseloading teams, including safeguarding, public health, project management, and leadership
**Exclusion criteria**
Midwives who work in the standard model of maternity care
OR have worked in caseloading teams at different maternity providers
OR are Maternity Support Workers (these practitioners having different professional roles and responsibilities)
OR decline to provide their informed consent

### Recruitment of childbearing women and data collection

Recruitment and data collection with midwives and childbearing women is concurrent. Data collection involves conducting semi-structured interviews with both participant groups. Literature available online, including research, reports, policies, and guidance is also being analyzed to assist with theory development.

In order to reach a wide audience, the study has been publicized in partnership with key stakeholders across corporate social media pages. A Facebook™ page has been developed, which is being used to advertise the study and provide a space to post relevant documents, such as the Participant Information Sheet (PIS) and posters. The Facebook™ page has been used as a form of communication between the research team and potential participants. Women who are interested in taking part contact the research team via private messages on Facebook, via email, or by telephone. The research team then contact potential participants to discuss the study and to confirm eligibility.

Midwives and key stakeholders have been integral to the success of recruitment. Women have been provided with study information packs and those interested in taking part have either contacted the research team or have provided their contact details to staff and the research team have made contact. The lead researcher, CC, has also attended parenting groups facilitated by stakeholders on Zoom™ to inform people about the study.

Interviews are being conducted remotely by CC. The PIS is explicit about CC being a midwife and that she will conduct the research interviews. Interviews are being conducted using video-conferencing software provided by Microsoft Teams™ or Zoom™, via email, WhatsApp™, Facebook Messenger™, or by telephone. Interviews by telephone or WhatsApp™, have been conducted using a study specific mobile phone. Interviews via all other platforms are being conducted on a study-specific computer provided by Bournemouth University (the study sponsor).

Adopting a flexible approach to data collection was influenced by the public. Women highlighted the importance of being able to choose which platform they completed their research interview on, and whether the interview was conducted via telephone or on a personal computer.

Interviews follow a semi-structured interview guide exploring the context of women’s lives. In addition, questions explore women’s perception of the midwives’ public health role and their experience of care. Through ConGT methods of concurrent data collection, analysis, constant comparisons, and theoretical sampling, new questions are developed and posed during interviews and at subsequent interviews with new participants. Before and after each interview the lead researcher records thoughts and insights in a reflective journal to inform theory development.

### Recruitment of midwives and data collection

The study has been advertised using posters throughout the maternity department and discussed at relevant departmental meetings. Recruitment emails have also been sent. Midwives either contact the lead researcher or provide their details and are contacted by the research team.

Interviews are being conducted remotely in a format of the participants’ choice, as described above. Interviews follow a semi-structured interview guide exploring midwives understanding of the SDH and their public health role. Prompts and new questions are developed and posed during interviews and at subsequent interviews.

**Figure 2:**
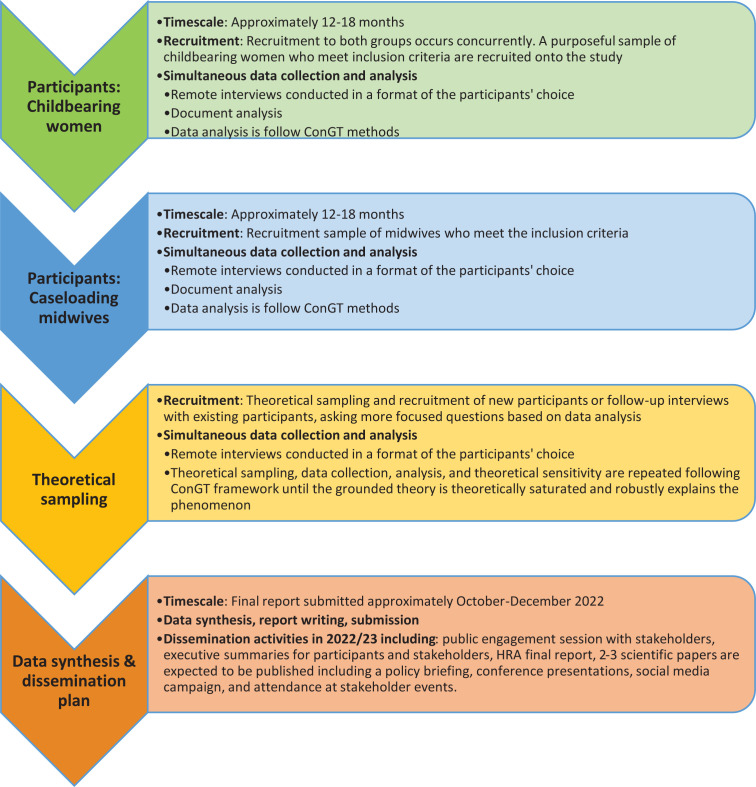
Study timeline

Figure 2 provides a detailed timeline of the recruitment period for each participant group, data analysis, synthesis, and dissemination activities once the final report is complete.

### Reflexivity of the study team

Personal and professional identities and philosophies have undoubtedly influenced preconceptions and impact the dynamics of the interview space. They also influence the way in which questions are posed, the information participants feel comfortable to share, data analysis, and the co-creation of knowledge. ConGT emphasizes participants' implicit meanings and researcher’s constructions of reality and accounts for the influence of the social contexts in which they are constructed^31,32^. Moreover, ConGT acknowledges the influence of the researcher and encourages reflexivity. Reflexive memos have been kept to ensure all preconceptions, actions, and interpretations are critically analyzed and peer-reviewed by the supervisory team. This is consistent with the principles of ConGT and upholds transparency, authenticity, and rigor^32^.

### Consent process and confidentiality

Consent is confirmed and documented electronically. Participants complete an online consent form prior to any data collection. They are sent a link to the consent form after the research team has confirmed eligibility. The consent form involves a set of simple, self-assessment questions which confirm participants' understanding. Each statement must be answered in order to progress to the next. Participants select either ‘yes’ or ‘no’, to indicate their agreement, if they select ‘no’ to any mandatory statement they will be redirected to the end of the form, which thanks them for their time and provides instructions about exiting the page, thereby declining their informed consent. The end of the form also provides hyperlinks to the PIS and the contact details of the research team. An electronic signature is collected at the end of the form, confirming whether the participant agrees to provide their informed consent or not. An option of ‘yes, I do provide consent’ or ‘no, I do not wish to provide consent’ confirms whether they agree to participate or not. Both the HRA and the Medicines and Healthcare Products Regulatory Agency^33^, promote the use of e-consent procedures in studies with minimal risk of harm. The consent form supports the following accessibility functions: text-to-speech and adjustable font size, which can be selected by participants to aid understanding and completion of the form. Consent is verbally re-confirmed with each participant prior to any data collection.

All study procedures are compliant with the Data Protection Act^34^ and the European General Data Protection Regulations (GDPR)^35^. Each participant is allocated a unique study identifier and a pseudonym, which will be used to protect confidentiality in future publications. Job titles held by the midwives will not appear in final reports to protect confidentiality. Participants’ personal identifiable information will be held securely and separately from other study documentation by the study sponsor. Only the research team have access to the complete data set. There are also specific circumstances when the research team are required to share information with other named professionals. For instance, if participants are at risk of harming themselves or others, or if they disclose concerns about the practice of a healthcare professional. The actions required to protect participants’ wellbeing are explained in the PIS, the consent form, and re-confirmed at the beginning of each interview. The research team acknowledge this may impact what participants feel comfortable to share and the impact this may have on the development of theory.

### Sampling technique and sample size

In the early phases of data collection, a purposeful sampling strategy was employed. Participants were recruited because they were representative of the target population groups. Opportunistic and snowball strategies also identified additional participants^32^. After an initial period of data analysis, the sampling strategy took the form of theoretical sampling, which is a method unique to grounded theory research^32^. Theoretical sampling focuses on the generation or collection of data that will theoretically saturate the emergent, core category or categories, and the related themes^32^. This involves a process of data collection for theory development, whereby the researcher jointly collects, codes, and analyses data, and then decides what to collect next, how, and what questions to ask future participants.

Sampling will cease at the point we reach theoretical saturation or data adequacy, that is, when categories are robust, rich, and show variation. The resulting theory should be rich, logical, and without gaps in explanation. Practical restrictions impacting sampling and recruitment such as time and financial restrictions may also influence the duration of sampling. However, based on previous reports of GT studies^36^, we anticipate completing between 20–40 interviews to achieve saturation.

### Ethical considerations

This study was granted regulatory and ethical approval on 29 May 2020 by South Central – Oxford C Research Ethics Committee (Ref: 20/SC/0183; IRAS 262369) and received HRA approval on 20 July 2020. There were no substantial changes to the study design reported in this protocol after commencement of the study. The study has also received Sponsorship from Bournemouth University and was prospectively registered on the Clinicaltrials.gov repository on 21 August 2020 (NCT04524286). Data collection was significantly delayed due to the impact of the COVID-19 pandemic on non-COVID related research activity across the NHS. The study commenced recruitment in November 2020 during the second national lockdown in England.

Participants can withdraw their consent until the point at which data is anonymized, without reason, and their normal care will not be affected. Data will be kept securely on a Sponsor approved device for five years following the end of the study. The PIS is explicit about the use and storage of participants’ data in line with GDPR.

Participants are remunerated for their time and contribution to the research by way of a £15 voucher, which is sent electronically or by post after participants complete their interview. This was an important design element, as it is crucial to recognize the value and significance of women’s time and contribution to this study. These involvement payments are being met by funds held by the study Sponsor.

The research team have a statutory duty of care to safeguard and protect the public in accordance with their professional code of practice^37^. A robust hazard log has been developed to mitigate against anticipated and unexpected risks.

### Data analysis

Data analysis involves the initial and focused coding procedures developed by Charmaz^31,32^. Data is initially coded line-by-line using ‘*in vivo*’ codes, these are labelled using words or short phrases from the participants own language. Alongside ‘*in vivo*’ codes, a comparative study of events occurring across the data is also being conducted. Here, similar events are being compared with one another to look for connections in the data. Data is analyzed for context, compared with one another, and then coded. Coding in this way assists in identifying subtle patterns and properties of the emerging categories. Comparing opposing events in the data provides further theoretical insights.

Focused coding procedures involve making sense of the data by grouping initial codes together into coherent categories. Constant comparison techniques are also being used throughout. Here data and focused codes are compared and contrasted with each other to understand similarities and differences in how participants understand their situations, actions, and social contexts^32^. Making constant comparisons helps to move beyond description to thinking more analytically and increases theoretical sensitivity. Theoretical sensitivity facilitates insights into what is meaningful and significant for the emerging theoretical framework and also helps to develop the properties and dimensions of categories and subcategories. As our theoretical sensitivity develops, we become better equipped to answer the question: ‘What is happening in the data?’.

## DISCUSSION

### Limitations

Conducting research during the COVID-19 pandemic has created multiple issues and this study is not free of limitations. For example, recruitment so far has been successful but our heavy reliance on digital technologies has created barriers for women who experience digital exclusion. Digital exclusion disproportionately impacts people from low-income backgrounds and restricts equitable access and engagement with research^38^. Simultaneously, women accessing maternity services are generally comfortable with technology as it has been part of their adult lives or they are ‘digital natives’, for whom digital technology has been a normal part of their entire lives^39^. In addition, public engagement activities concluded this to be an appropriate recruitment strategy, given the circumstances and pressures created by the COVID-19 pandemic.

Additional limitations include that participants are aware the study is examining their local service and this may impact responses. However, during preliminary data analysis, participants revealed positive and negative experiences. To increase rigor these insights will be compared using constant comparative methods to inform theory development. In addition, this study focuses on community-based, MCoC teams in urban contexts. Research examining rural models and innovations in the use of digital technologies with MCoC models should also be conducted as these contexts will reveal additional unique insights.

## CONCLUSIONS

With health and social inequities continuing to rise in England, the public health role of MCoC caseloading midwives is an important feature in helping to tackle disparities. The specific actions caseloading midwives take to advance equity in deprived areas is not well defined in a UK context. This study will provide new, mid-level theory from which future research hypotheses can be proposed and tested, in addition to expanding our existing understanding about the mechanisms underpinning the benefits of MCoC in different contexts. This research is timely and will provide important evidence to inform maternity policy, midwifery education, practice, and research.

## Data Availability

The data supporting this research are available from the authors on reasonable request.

## PROTOCOL REGISTRATION

Clinicaltrials.gov: NCT04524286. Submitted on 21.08.2020 Protocol version: The Mi-CARE Study. v1.3. 19.03.2021
